# Synthesis and Biological Analysis of Thiotetra(ethylene glycol) monomethyl Ether-Functionalized Porphyrazines: Cellular Uptake and Toxicity Studies

**DOI:** 10.1155/2008/391418

**Published:** 2007-10-01

**Authors:** Sangwan Lee, Benjamin J. Vesper, Hong Zong, Neal D. Hammer, Kim M. Elseth, Anthony G. M. Barrett, Brian M. Hoffman, James A. Radosevich

**Affiliations:** ^1^Department of Chemistry, Northwestern University, 2145 Sheridan Road, Evanston, IL 60208, USA; ^2^Center for Molecular Biology of Oral Diseases, College of Dentistry, University of Illinois at Chicago, 801 S. Paulina Street, Chicago, IL 60612, USA; ^3^Jesse Brown VAMC, 820 South Damen Avenue, Chicago, IL 60612, USA; ^4^Department of Chemistry, Faculty of Natural Sciences, Imperial College London, Faculty Building, South Kensington Campus, London SW7 2AZ, UK

## Abstract

The porphyrazines (pzs), a class of porphyrin analogues, are being investigated for their potential use as tumor imaging/therapeutic agents. We here examine six peripherally-functionalized M[pz(${\text{A}}_{n}{\text{B}}_{4\text{-}n}$)] pzs with n=4, 3, or 2 (in a *trans* conformation) and M = ${\text{H}}_{2}$ or Zn, where A is an ${\left[\text{S}{\left({\left({\text{CH}}_{2}\right)}_{2}\text{O}\right)}_{4}\text{Me}\right]}_{2}$ unit and B is a fused β,β′-diisopropyloxybenzo group. Cell viability/proliferation assays and fluorescence microscopy were carried out in both tumor and normal cells. Dark toxicity studies disclosed that four of the compounds exhibited toxicity in both normal and tumor cells; one was nontoxic in both normal and tumor cells, and one was selectively toxic to normal cells. Additionally, three of the pzs showed enhanced photo-induced toxicity with these effects in some cases being observed at treatment concentrations of up to ten-fold lower than that needed for a response in Photofrin. All six compounds were preferentially absorbed by tumor cells, suggesting that they have potential as in vitro diagnostic agents and as aids in the isolation and purification of aberrant cells from pathological specimens. In particular, two promising diagnostic candidates have been identified as part of this work.

## 1. INTRODUCTION

**Scheme 1:**
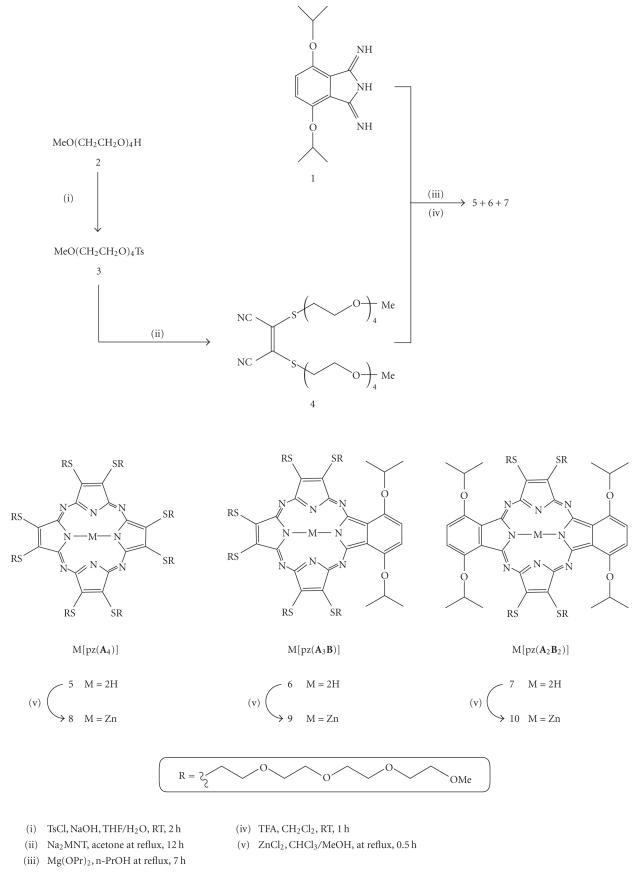
Synthetic route to porphyrazines **5**–**10**.

Porphyrin antitumor agents have been widely studied in recent years, especially in the area of photodynamic therapy (PDT), where the development of the clinically used porphyrin, haematoporphyrin derivative (HpD, or Photofrin), has garnered significant interest [1, 2]. However, limitations in the design, synthesis, and biological properties of Photofrin have led to the development of many second-generation PDT agents, including a number of porphyrin derivatives [3–9]. One such class of compounds being investigated as an alternative is the porphyrazines (pzs), porphyrin analogues in which the *meso* (CH) groups are replaced by nitrogen atoms linking the pyrrole rings (Scheme 1, Section 3), thereby resulting in compounds with chemical and physical properties distinct from those of the porphyrins [10]. The presence of the *meso*-nitrogen atoms results in the pzs possessing intense long-wavelength absorbance and emission bands, photophysical properties that are intrinsically superior to those of the porphyrins. Furthermore, the porphyrazines are prepared by the templated cyclization of maleonitrile derivatives, while porphyrins are synthesized by the condensation of aldehyde and pyrrole derivatives. This difference allows pzs to be readily prepared with S, N, or O heteroatoms attached to the macrocycle core, while porphyrins cannot. These heteroatom substituents enable the photophysical properties of the pzs to be tuned such that they exhibit optimal near-IR absorbance and emission in the 700–900 nm window, and allow for tuning of their singlet oxygen quantum yields from essentially *off* to *on*—characteristics which make the pzs attractive candidates for biomedical applications [11–14].

Because of the novelty of these compounds, initial biocompatibility studies involving the pzs have focused upon determining how specific pz structures selectively alter biological functions [15, 16], leading to the establishment of “structure-function rules,” which will ultimately be used to predict future biologically active pzs. To this end, we recently reported the biological activity of a suite of three anionic H_2_[pz(**A **_n_ **B** ${}_{4\text{-}n}$)] pzs, in which n=4, 3, or 2 (*trans*), **A** was an [S-R]_2_ unit with $\text{R}={\left({\text{CH}}_{2}\right)}_{3}{\text{CO}}_{2}{}^{-}$, and **B** was a fused β,β′-dialkoxybenzo group. These three anionic compounds showed a systematic increase in toxicity as n (and in turn, the number of R groups) was increased, and one pz (n=2) was found to be selectively toxic to tumor cells. Herein we similarly describe the biocompatibility of a group of M[pz(A_n_ B${}_{4\text{-}n}$)] pzs, where M = H_2_ or Zn, n=4, 3, or 2 (*trans*), **A** is an [S-R]_2_ unit with R = [(CH_2_)_2_O]_4_Me, and **B** is again a fused β,β′-dialkoxybenzo group (**5**–**10**, Scheme 1).^1^

As with the aforementioned anionic porphyrazines, the pzs examined here, with thiotriethoxy(ethoxy)methyl groups appended to their periphery, allow us to study the biological effect of a specific functional group. By varying n, we can simultaneously vary the core pz structure and the number of appended R groups, resulting in each compound possessing slightly different chemical and physical properties, thereby impacting the biological effects of each.

This study also marks the first biocompatibility experiments involving metallated pzs. As previously reported, the singlet oxygen quantum yields (*Φ*_Δ_) of the free-base (H_2_) pzs increase systematically as n is decreased; the same trend is true for metallated pzs, but the presence of Zn in the pz core increases the value of *Φ*_Δ_ approximately five-fold over that of the analogous H_2_ pzs [12]. Herein we report the concentration/time-dependent cytotoxicity and phototoxicity measurements, and present confocal fluorescence microscopic visualization, for pzs **5**–**10** in A549 tumor and WI-38 VA13 normal cells.

## 2. MATERIALS AND METHODS

### 2.1. Chemical reagents and instrumentation

 All chemicals were purchased from Aldrich Chemical Company “Wis, USA” and used as received. Baxter silica gel (60 Å; 230–400 mesh) was used for column chromatography. A Hewlett-Packard HP8452A diode-array spectrophotometer was used to record electronic absorption spectra, and electronic emission spectra were recorded using a Photon Technology International QM2 fluorescence spectrometer. ^1^H and ^13^C NMR spectra were obtained using a Varian Inova 500 MHz spectrometer. Electrospray ionization mass spectra (ESI-MS) were recorded using a Finnegan LCQ Advantage mass spectrometer, and matrix-assisted laser desorption ionization-time of flight mass spectra (MALDI-TOF-MS) were recorded using a PE Voyager DE-Pro MALDI-TOF mass spectrometer; *α*-cyano-4-hydroxycinnamic acid was used as the matrix for MALDI-TOF experiments.

### 2.2. Porphyrazine synthesis

Disodium 1,2-dicyano-1,2-ethenedithiolate (Na_2_MNT) [17] and 1-imino-4,7-bis(1-methylethoxy)-1H-isoindolin-3-amine (diiminoisoindoline, **1**) [11] were prepared as previously reported. During the purification of each pz, distinct bands were observed and pooled together during column chromatography; collected fractions were monitored by UV-visible spectroscopy. Each pz was prepared as a 5 mM working stock solution in dimethyl sulfoxide (DMSO) for use in the tissue culture experiments.

### 2.3. 2-*{*2-[2-(2-Methoxyethoxy)ethoxy]ethoxy*}* ethyl-4-methylbenzenesulfonate (3)

Sodium hydroxide (12 g, 0.3 mol) dissolved in water (55 mL) and tetra(ethylene glycol) monomethyl ether (**2**) (41.7 g, 0.2 mol) in THF (55 mL) were placed in a flask, and the mixture was stirred and cooled in an ice bath. To the mixture was added dropwise p-toluenesulfonyl choride (41.9 g, 0.22 mol) in THF (65 mL) over 30 minutes with continuous stirring and cooling of the mixture below 5°C. The solution was stirred an additional 2 hours at *∼*0–5°C, at which time the mixture was poured into ice water (200 mL) and extracted twice with CH_2_Cl_2_ (200 mL). The combined organic extract was washed twice with water and once with a saturated NaCl solution (brine), and then dried over Na_2_SO_4_. After evaporation of the solvent, the resulting residue was purified by chromatography on silica gel (4% MeOH in CH_2_Cl_2_ eluant) to yield the tosylate **3** (63 g, 87%) as a colorless viscous oil: ^1^H NMR (500 MHz, CDCl_3_) *δ* 2.43 (s, 3H, ArCH_3_), 3.35 (s, 3H, OCH_3_), 3.52 (t, 2H, CH_2_), 3.50−3.60 (m, 10H, CH_2_), 3.67 (t, 2H, CH_2_), 4.14 (t, 2H, CH_2_), 7.33 (d, 2H, ArH), 7.78 (d, 2H, ArH); ^13^C NMR (125 MHz, CDCl_3_) 21.8, 59.1, 68.8, 69.4, 70.6, 70.8, 71.2, 71.6, 71.9, 72.2, 128.1, 130.0, 133.1, 145.0; ESI-MS (*m/z*) calculated for C_16_H_27_O_7_S [M + H]^+^ 363.44, found 363.

### 2.4. bis*{*2-*{*2-[2-(2-Methoxyethoxy)ethoxy]ethoxy*}*ethylthio*}*maleonitrile (4)

Na_2_MNT (7.45 g, 0.04 mol) was treated with two equivalents of **3** (30.45 g, 0.084 mol) in acetone (80 mL) at reflux under a nitrogen atmosphere for 12 hours. The solvent was then removed under reduced pressure, and the resulting residue was purified by chromatography on silica gel (4% MeOH in CH_2_Cl_2_ eluant) to yield **4** (13.40 g, 64%) as a viscous yellow oil: ^1^H NMR (500 MHz, CDCl_3_) *δ* 3.30 (t, 4H, CH_2_), 3.56 (s, 6H, OCH_3_), 3.50 (t, 4H, CH_2_), 3.53 (t, 4H, CH_2_), 3.58 (t, 4H, CH_2_), 3.62 *∼* 3.64 (m, 8H, CH_2_), 3.65 (t, 4H, CH_2_), 3.74 (t, 4H, CH_2_); ^13^C NMR (125 MHz, CDCl_3_) 21.8, 59.1, 68.8, 69.4, 70.7, 70.8, 71.3, 71.7, 72.0, 73.2, 128.1; ESI-MS (*m/z*) calculated for C_22_H_39_N_2_O_8_S_2_ [M + H]^+^ 523.69, found 523.

### 2.5. 2,3,7,8,12,13,17,18-octakis*{*2-*{*2-[2-(2-Methoxyethoxy)ethoxy]ethoxy*}*ethylthio*}*-21H,23H-porphyrazine (5)

Mg turnings (0.1 g, 4 mmol) and I_2_ (0.01 g, $4\times 10^{-5}$ mol) in n-PrOH (50 mL) were heated at reflux for 24 hours under N_2_ to prepare Mg(OPr)_2_. MNT[S(C_2_H_4_O)_4_Me]_2_ (**4**) (5.23 g, 0.01 mol) was added, and the reaction was heated at reflux for 7 hours. The yellow reaction mixture gradually turned green. After 2 hours, the reaction mixture was a deep blue color. Heating and stirring were continued for a total 7 hours after which the solvent was removed under reduced pressure. The resulting residue was dissolved in CH_2_Cl_2_ (20 mL). TFA (2 mL) was slowly added, and the solution was stirred for 1 hour. After dilution in CH_2_Cl_2_ (100 mL), the mixture was washed 4 times with a large amount of water to remove any residual TFA, dried over Na_2_SO_4_, and rotary evaporated. The resulting residue was purified via column chromatography (4% MeOH in CH_2_Cl_2_ eluant) to produce **5** (628 mg, 12% yield) as a dark blue solid: UV-vis (CH_2_Cl_2_) $\lambda_{\max}$(log *ɛ*) 360 (4.66), 502 (2.23), 640 (3.10), 710 (3.99) nm; ^1^H NMR (500 MHz, CDCl_3_) *δ*-1.13 (br s, 2H, NH), 3.32 (s, 24H, –OCH_3_), 3.40 (t, 16H, CH_2_), 3.55 *∼* 3.59 (m, 64H, CH_2_), 3.65 (t, 16H, CH_2_), 3.99 (t, 16H, CH_2_), 4.28 (t, 16H, CH_2_); ^13^C NMR (125 MHz, CDCl_3_) 34.76, 59.23, 70.68, 70.72, 70.74, 70.77, 71.11, 72.09, 140.56; MALDI-TOF-MS (*m/z*) calculated for C_88_H_155_N_8_O_32_S_8_ [M + H]^+^ 2093.72, found 2093.66; calculated for C_88_H_154_N_8_NaO_32_S_8_ [M + Na]^+^ 2115.71, found 2116.48.

### 2.6. *{*2-*{*2-[2-(2-Methoxyethoxy)ethoxy]ethoxy*}*ethylthio*}*porphyrazines 6 and 7

Mg turnings (0.3 g, 12 mmol) and I_2_ (0.03 g, $1.2\times 10^{-4}$ mol) in n-PrOH (150 mL) were heated at reflux for 24 h under N_2_ to prepare Mg(OPr)_2_. MNT[S(C_2_H_4_O)_4_Me]_2_ (**4**) (5.23 g, 0.01 mol) and diiminoisoindoline (**1**) (5.23 g, 0.02 mol) were added, and the reaction was heated at reflux for 7 hours, during which time the solution went from orange to blue-black. The solvent was removed under reduced pressure, and the residue was dissolved in CH_2_Cl_2_ (30 mL). TFA (3 mL) was slowly added, and the solution was stirred for 1 hour. After dilution in CH_2_Cl_2_ (200 mL), the mixture was washed 4 times with a large amount of water to remove any residual TFA, dried over Na_2_SO_4_, and rotary evaporated. The resulting residue was purified via column chromatography (4% MeOH in CH_2_Cl_2_ eluant) to yield **6** (260 mg, 4.3%) as a dark blue solid and **7** (477 mg, 6.2%) as a dark green solid, as well as trace amounts of **5**.

### 2.7. 19,22-bis(1-Methylethoxy)-4,5,9,10,14,15-hexakis*{*2-*{*2-[2-(2-methoxyethoxy)ethoxy]-ethoxy*}*ethylthio*}*-23H,25H-porphyrazine (6)

UV-vis (CH_2_Cl_2_) $\lambda_{\max}$ (log *ɛ*) 350 (4.61), 658 (3.82), 702 (4.35), 744 (sh) nm; ^1^H NMR (500 MHz, CDCl_3_) *δ*-0.20 (br s, 2H, NH), 2.02 (d, 12H, –CHMe_3_), 3.69 (s, 18H, OCH_3_), 3.76 *∼* 3.79 (m, 48H, CH_2_), 3.84 *∼* 3.87 (m, 24H, CH_2_), 4.02 (t, 4H, CH_2_), 4.17 (t, 4H, CH_2_), 4.19 (t, 4H, CH_2_), 4.39 (t, 4H, CH_2_), 4.46 (t, 4H, CH_2_), 4.51 (t, 4H, CH_2_), 5.49 (sept, 2H, –CHMe_2_), 7.87 (s, 2H, ArH);^13^C NMR (125 MHz, CDCl_3_) 22.3, 22.4, 22.7, 22.9, 29.9, 34.7, 34.8, 34.9, 36.5, 59.2, 70.6, 70.7, 70.8, 71.1, 71.2, 72.1, 72.7, 119.6, 126.5, 138.4, 138.8, 141.9, 150.5; ESI-MS (*m/z*) calculated for C_80_H_133_N_8_O_26_S_6_ [M + H]^+^ 1815.36, found 1815.70; calculated for C_80_H_132_N_8_NaO_32_S_8_ [M + Na]^+^ 1837.34, found 1837.76.

### 2.8. 1,4,13,16-tetrakis(1-Methylethoxy)-8,9,20,21-tetrakis*{*2-*{*2-[2-(2-methoxyethoxy)ethoxy]-ethoxy*}*ethylthio*}*-25H,27H-dibenzo[b,l]porphyrazine (7)

UV-vis (CH_2_Cl_2_) $\lambda_{\max}$ (log *ɛ*) 342 (4.80), 654 (5.41), 712 (2.20), 796 (4.31) nm; ^1^H NMR (500 MHz, CDCl_3_) *δ*-0.44 (br s, 2H, NH), 2.26 (d, 24H, CHMe_2_), 3.91 (s, 24H, OCH_3_), 3.93 *∼* 3.96 (m, 24H, CH_2_), 4.08 *∼* 4.14 (m, 16H, CH_2_) 4.18 (t, 8H, CH_2_), 4.24 (t, 8H, CH_2_), 4.77 (t, 8H, CH_2_), 5.28 (sept, 4H, CHMe_2_), 7.56 (s, 4H, ArH); ^13^C NMR (125 MHz, CDCl_3_) 22.7, 23.0, 35.1, 59.1, 70.6, 70.7, 70.8, 71.0, 71.1, 71.3, 72.0. 72.5, 118.5, 128.3, 138.7, 149.8; ESI-MS (*m/z*) calculated for C_72_H_111_N_8_O_20_S_4_ [M + H]^+^ 1536.96, found 1536.63; calculated for C_72_H_110_N_8_NaO_20_S_4_ [M + Na]^+^ 1558.95, found 1558.70.

### 2.9. Zn porphyrazines 8, 9, and 10

The three metallated pzs were each prepared analogously to zinc pzs previously described [11]. A solution of zinc chloride (20.4 mg, 0.15 mmol) in methanol (10 mL) was added to a solution of metal-free porphyrazine (0.05 mmol) in CH_2_Cl_2_ (30 mL). The reaction mixture was heated to reflux for 30 minutes or until zinc insertion was completed, as monitored by spectrophotometry. The reaction mixture was diluted with CH_2_Cl_2_ (60 mL), washed with water to remove excess zinc chloride, dried with Na_2_SO_4_, and evaporated to dryness under vacuum. The resulting residue was purified by chromatography on silica gel (4% MeOH in CH_2_Cl_2_ eluant) to yield the desired Zn porphyrazine in quantitative yield.

### 2.10. 2,3,7,8,12,13,17,18-octakis*{*2-*{*2-[2-(2-Methoxyethoxy)ethoxy]ethoxy*}*ethoxy*}* ethylthio-porphyrazine zinc(II) (8)

UV-vis (CH_2_Cl_2_) $\lambda_{\max}$ (log *ɛ*) 378 (4.62), 672 (4.67) nm; MALDI- TOF-MS (*m/z*) calculated for C_88_H_153_N_8_O_32_S_8_Zn [M + H]^+^ 2157.12, found 2157.32; calculated for C_88_H_152_N_8_NaO_32_S_8_Zn [M + Na]^+^ 2179.10, found 2179.24.

### 2.11. 19,22-bis(1-Methylethoxy)-4,5,9,10,14,15-hexakis*{*2-*{*2-[2-(2-methoxyethoxy)ethoxy]-ethoxy*}*ethylthio*}*-23H,25H-porphyrazine zinc(II) (9)

UV-vis (CH_2_Cl_2_) $\lambda_{\max}$ (log *ɛ*) 362 (4.60), 618(sh), 676 (4.69), 706(sh) nm; ESI-MS (*m/z*) calculated for C_80_H_131_N_8_O_26_S_6_Zn [M + H]^+^ 1878.73, found 1878.60.

### 2.12. Zn(II) 1,4,13,16-tetrakis(1-methylethoxy)-8,9,20,21-tetrakis*{*2-*{*2-[2-(2-methoxyethoxy)-ethoxy]ethoxy*}*ethylthio*}*-25H,27H-dibenzo[b,l]porphyrazine (10)

UV-vis (CH_2_Cl_2_) $\lambda_{\max}$ (log *ɛ*) 352 (4.64), 666 (4.73), 761(4.64) nm; ESI-MS (*m/z*) calculated for C_72_H_109_N_8_O_20_S_4_Zn [M + H]^+^ 1600.34, found 1599.63.

### 2.13. Cell culture and cell lines

All media and supplements were purchased from Invitrogen “Calif, USA” except where noted. Phosphate-buffered saline (PBS) solution was prepared by adding Na_2_HPO_4_ •7 H_2_O (54.0 g), KH_2_PO_4_ (5.0 g), KCl (5.0 g), and NaCl (200.0 g) to 25 L of distilled water. A 2.0 g/L solution of 3-[4,5-dimethylthiazol-2-yl]-2,5-diphenyltetrazolium bromide (MTT) in PBS was prepared and sterile filtered (0.2 *μ*m pore size) prior to use in the cytotoxicity assays. Haematoporphyrin derivative (HpD, or Photofrin; QLT Phototherapeutics, Inc. “Vancouver, BC, Canada” ) was used as a positive control in the phototoxicity assays; concentrations of HpD were calculated using an assigned molecular weight of 600.

The following cell lines were utilized in this study: A549 (human lung adenocarcinoma), WI-38 VA13 (SV40 transfected fibroblast-like human embryonic cell line), five human head and neck squamous cell carcinomas (SCC016, SCC040, SCC056, SCC114, SCC116), and three human breast adenocarcinomas (BT-20, T-47D, and MCF-7). All cell lines were obtained from American Type Culture Collection “Va, USA.” The A549 and T-47D cell lines were maintained in RPMI 1640 media supplemented with 10% fetal calf serum heat inactivated at 56°C for 30 minutes, 2 mM L-Glutamine, 100 *μ*g/mL Streptomycin, 100 U/mL Penicillin, and 2.5 mcg/mL Amphotericin B solution. All other cell lines were maintained in minimum essential medium (MEM) with Earle’s salts supplemented with 10% fetal calf serum heat inactivated at 56°C for 30 minutes, 2 mM L-Glutamine, 100 *μ*g/mL Streptomycin, 100 U/mL Penicillin, and 2.5 mcg/mL Amphotericin B solution, as well as 100 *μ*M MEM nonessential amino acids and 1 mM Sodium Pyruvate (CellGro, Inc. “Va, USA”) in distilled water. Cells were grown at 37°C in a humidified atmosphere containing 5% CO_2_.

### 2.14. Cytotoxicity assays

Cells were seeded (100 *μ*L) into 96-well microtiter plates and grown until they were approximately 70% confluent. The plates were then treated in the dark (to avoid photosensitized killing) with the appropriate concentration of pz or a volume of DMSO equivalent to the volume of compound added at these concentrations. Pz and DMSO solutions were added as 100 *μ*L aliquots to the original media in the well. The subsequent 200 *μ*L of media was thoroughly mixed, after which time 100 *μ*L was removed, resulting in a final volume of 100 *μ*L in each well; final pz/DMSO concentrations ranged from 50–1.56 *μ*M. The media were removed at designated time points (24, 48, 72, 96, or 120 hours), and 100 *μ*L of MTT/PBS solution was added to each well. The plates were then incubated for an additional 5 hours at 5% CO_2_ and 37°C. During this time, the tetrazolium ring of the MTT molecules is cleaved by the mitochondrial dehydrogenases of viable cells, resulting in the formation of purple formazan crystals. Following incubation, the supernatant was decanted, and 100 *μ*L of DMSO was added to each well to dissolve any formazan crystals. The absorbance was then read at 540 nm for each well. Each data point represents the average of at least four microtiter wells for each plate, and at least three independent trials were carried out for each experiment. Individual trials were normalized and averaged such that the final reported values represent a minimum of 12 independent values for each reported condition for each cell line.

### 2.15. Phototoxicity assays

Two identical 96-well plates were seeded with A549 and WI-38 VA13 cells grown to *∼*70% confluency as described above. The cells were then treated with the desired concentration of pz or HpD and incubated for additional 4 hours. Aluminum foil was then wrapped around the sides and top of the first plate, leaving the bottom of the plate uncovered in order to enable light penetration. The second plate was completely wrapped in aluminum foil to inhibit light from reaching the cells; this plate served as the dark control. The two plates were then placed on top of a standard X-ray illuminator (consisting of four 15 W bulbs, *∼*3600 total lumens) and exposed to 10 minutes of light. Following the light treatment, the plates were placed back into the incubator for 24 hours, after which time MTT cytotoxicity assays were performed.

### 2.16. Imaging assays

A549 and WI-38 VA13 cells were plated onto sterilized glass coverslips in 60×15 mm dishes and grown at 37°C in a humidified atmosphere containing 5% CO_2_. Upon reaching 60–80% confluency, the cells were treated with the desired pz at 25 *μ*M and incubated in the dark under the same conditions for an additional 4 hours. Negative controls were prepared by treating cells with an amount of DMSO equivalent to that of the 25 *μ*M pz sample.

Confocal microscopic images of the red pz emission were obtained at room temperature with a Zeiss 510 LSM confocal microscope. Following the 4-hour incubation period of the cells with the pz, the supernatant was decanted and the cells were washed twice with PBS. The washed coverslips were then inverted onto microscope slides, and the cells were imaged live in PBS. Cells were excited with an argon-ion laser line at 488 nm, and fluorescence was detected with a long-pass 505 nm filter. All images were taken using the same detector gain and amplitude settings.

## 3. RESULTS AND DISCUSSION

### 3.1. Pz synthesis and properties

The six compounds examined in this study are of the form M[pz(**A**_n_ **B** ${}_{4\text{-}n}$)], where M = H_2_ or Zn, **A** is [S((CH_2_)_2_O)_4_CH_3_]_2_, and **B** is a fused 4,7-bis(isopropyloxy)benzo group, with n=4 (**5**, **8**), n=3 (**6**, **9**), and the *trans* form of n=2 (**7**, **10**). Scheme 1 shows the reaction scheme utilized to prepare the pzs.

In order to attach the desired tetra(ethylene glycol) monomethyl ether, (CH_2_CH_2_O)_4_Me, functional group onto the periphery of a pz, it was first necessary to prepare 2-*{*2-[2-(2-methoxyethoxy)ethoxy]ethoxy*}*ethyl-4-methylbenzenesulfonate (**3**) via tosylation of the commercially available tetra(ethylene glycol) monomethyl ether (**2**). MNT[S(C_2_H_4_O)_4_Me]_2_ (**4**) was then prepared by treating Na_2_MNT with two equivalents of **3** in acetone at reflux for 12 hours.

Magnesium-templated Linstead macrocyclization [18] of MNT[S(C_2_H_4_O)_4_Me]_2_ (**4**), followed by demetallation in trifluoroacetic acid and purification via column chromatography, led to the formation of the H_2_[pz(**A**_4_)], **5**, in 12% yield. The H_2_[pz(**A**_3_ **B**)] (**6**, 4% yield) and *trans*-H_2_[pz(**A**_2_ **B**_2_)] (**7**, 6% yield) pzs were prepared via a mixed cyclization of MNT[S(C_2_H_4_O)_4_Me]_2_ (**4**) and 1,3-diiminoisoindoline (**1**), reacted in a 2 : 1 stoichiometric ratio, and followed by demetallation with trifluoroacetic acid. Column chromatography was then used to separate and purify the two compounds. It is noted that the *cis*-H_2 _[pz(**A_2_B_2_**)] compound was not produced during this reaction, indicating that under these reaction conditions, the diiminoisoindoline (**1**) reacts preferentially with its cocyclization partner, **4**, resulting in only the *trans* form of the M[pz(**A_2_B_2_**)]. The yield of the *trans*-macrocycle was further improved by the use of 0.38 equivalents of Mg per mole of combined cyclization partners, and by heating the reaction mixture for 7 hours at reflux in n-propanol. The corresponding zinc analogues—**8**, **9**, and **10**—were prepared by reacting each of the free-base pzs in a solution of zinc chloride in CHCl_3_/MeOH at reflux for 30 minutes. All three compounds were obtained in quantitative yield.

**Figure 1:**
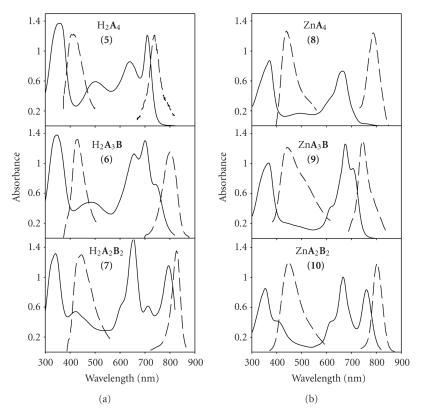
Absorbance (solid lines) and emission (dashed lines) spectra of H_2_[pz(**A**_n_ **B** ${}_{4\text{-}n}$)] and Zn[pz(**A**_n_ **B** ${}_{4\text{-}n}$)] pzs in CH_2_Cl_2_.

Each compound is freely soluble in DMSO. By utilizing different combinations of M (H_2_ or Zn) and n (4, 3, or 2, in a *trans* conformation), we can predictably vary the core macrocycle structure, resulting in different optical properties for each of the six pzs. Previous work has shown that the optical properties of the pzs are dependent upon M and n, but independent of R [10,11,19,20]. Figure 1 shows typical spectra obtained in CH_2_Cl_2_ for the six pzs presented here. All six compounds exhibit an intense B (Soret) band at *∼*350 nm with high extinctions (*∼*50,000 M^−1^cm^−1^), but each has a different Q-band region, depending upon M and n. For the free-base pzs, split Q-bands are observed for both n=3 and n=2 (*trans*), with H_2_[pz(**A**_3_ **B**)] exhibiting a maximum absorption of *∼*700 nm (*ɛ* *∼*45,000 M^−1^cm^−1^) and H_2_[pz(**A**_2_ **B**_2_)] having well-defined peaks at *∼*654 nm and 798 nm (*ɛ* *∼*50,000 M^−1^cm^−1^ for both), respectively; in contrast, the symmetrical H_2_[pz(**A**_4_)] (n=4) exhibits only a single Q-band with a maximum absorption at *∼*712 nm (*ɛ* *∼*35,000 M^−1^cm^−1^). As expected, similar Q-band patterns are observed in the three Zn[pz(**A**_n_ **B** ${}_{4\text{-}n}$)]pzs, but the peaks are shifted *∼*30 nm towards the blue. Zn[pz(**A**_4_)] has a single Q-band absorption at 672 nm (*ɛ* *∼*47,000 M^−1^cm^−1^); both Zn[pz(**A**_3_ **B**)] and Zn[pz(**A**_2_ **B**_2_)] have split Q-bands, with the former exhibiting a maximum absorption at 676 nm (*ɛ* *∼*50,000 M^−1^cm^−1^) and the latter having peaks at 666 nm (*ɛ* *∼*54,000 M^−1^cm^−1^) and 761 nm (*ɛ* *∼*45,000 M^−1^cm^−1^), respectively. All six compounds also exhibit dual fluorescence, as shown in Figure 1: for the free-base pzs with n=4, 3, and 2, short wavelength (uv) fluorescence is observed at $\lambda_{\max}$ = 410, 428, and 440 nm, respectively; and long wavelength (nir) fluorescence occurs at $\lambda_{\max}$ = 737, 800, and 827 nm, respectively; for the Zn pzs with n=4, 3, and 2, uv emission is observed at $\lambda_{\max}$ = 435, 444, and 440 nm, respectively; and nir emission occurs at $\lambda_{\max}$ = 713, 754, and 795 nm, respectively [10]. Both emissions can be generated with excitation wavelengths to the blue of *∼*400 nm, while only the nir luminescence is produced upon excitation to the red of *∼*450 nm. Unlike previously reported pzs in which broadening and aggregation were often observed [15, 16], the uv-visible spectra of **5–10** in DMSO looked nearly identical to the spectra obtained in CH_2_Cl_2_.

Earlier work with the pzs has also found that the singlet oxygen quantum yields (*Φ*_Δ_) for a series of M[pz(**A**_n_ **B** ${}_{4\text{-}n}$)], where **A** = [S-R]_2_ and **B** is a fused dialkoxybenzo group, are dramatically affected by both M and n, but are independent of the identity of the R group [12]. For a given M, the quantum yield is found to increase as the value of n is decreased. Therefore, the H_2_[pz(**A**_2_ **B**_2_)], **7**, has the highest quantum yield of the three free-base pzs (*Φ*_Δ_ *∼* 0.130), with **6** (*Φ*_Δ_ *∼* 0.026) and **5** (*Φ*_Δ_ *∼* 0.0074) have markedly lower values. Introducing zinc into the core of the pz results in significantly higher yields than those found in the analogous free-base compounds. Thus the Zn[pz(**A**_2_ **B**_2_)], **10**, possesses the highest singlet oxygen quantum yield (*Φ*_Δ_ *∼* 0.370) of the six compounds presented in this study, while the quantum yields for **9** (*Φ*_Δ_ *∼* 0.110) and **8** (*Φ*_Δ_ *∼* 0.037) are accordingly lower.

### 3.2. Quantitative in vitro effect of porphyrazines

**Figure 2:**
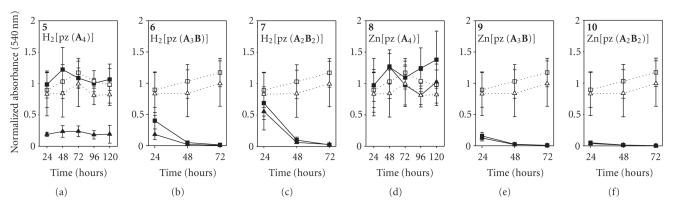
MTT time course plot of A549 (*□*) and WI-38 VA13 (Δ) cells exposed to 50 *μ*M pz. Dotted lines = control (cells + DMSO), solid lines = cells + pz.

MTT assays were used to test the proliferation/viability of A549 and WI-38 VA13 cells grown in the presence of compounds **5–10** over a 72-hour time period. Cells were initially treated with a pz concentration of 50 *μ*M, and MTT assays were performed at 24, 48, and 72 hours (Figure 2).

In order to confirm that any toxicity observed was due to the pzs and not the DMSO solvent, control cells were exposed to a volume of DMSO equivalent to the volume of pzs at 50 *μ*M; cells exposed to DMSO exhibited normal growth behavior. Unlike previously reported pzs which exhibited little cellular toxicity after a 24-hour exposure [15, 16], four of the six pzs tested here exhibited marked toxicity to both normal and tumor cells within the first 24 hours upon 50 *μ*M exposure. (We note here that the 50 *μ*M treatment dose was chosen because it corresponds to the approximate amount of HpD typically needed to observe a clinical response in patients undergoing PDT treatments.) Cells grown in the presence of H_2_[pz(**A**_2_ **B**_2_)], **7**, and the M[pz(**A**_3_ **B**)] pzs, **6** (M = H_2_) and **9** (M = Zn), showed moderate-to-large decreases in viability after 24 hours, while cells treated with Zn[pz(**A**_2_ **B**_2_)], **10**, were almost completely killed within the first 24 hours of exposure. An additional 24 hours of exposure to pzs **6**, **7**, and **9** resulted in both normal and tumor cells being nearly completely killed.

The proliferation/viability of cells exposed to the M[(**A**_4_)] pzs, **5** (M = H_2_) and **8** (M = Zn), contrasted sharply to that of the M[pz(**A**_3_ **B**)] and M[pz(**A**_2_ **B**_2_)] pzs. Compound **8** showed little-to-no toxicity in either cell line over the entire time course of 72 hours, while **5** was found to be selectively toxic to normal cells. Nearly 80% of the WI-38 VA13 normal cells were killed within 24 hours upon exposure to **5**, while no appreciable toxicity was seen for tumor cells exposed to **5** for 72 hours. The nontoxic behavior found with cells exposed to **5** (tumor only) and **8** (both tumor and normal) prompted the study of longer treatment times for these two compounds. Additional MTT assays were therefore carried out at 96 and 120 hours for both **5** and **8**. However, as shown in Figure 2, these longer exposure times did not result in any additional observed toxicity.

The selective killing of the normal cells upon exposure to **5** prompted us to test this particular compound against other tumor cell lines to determine if the resistance of the A549 cells was consistent across different tumor cell lines and types. Table 1 shows the percent viability of human breast adenocarcinomas (BT-20, T-47D, and MCF-7) and human head and neck squamous cell carcinomas (SCC016, SCC040, SCC056, SCC114, and SCC116) exposed to **5** at 50 *μ*M for 72 hours, relative to DMSO-treated control cells. In general, very little toxicity was observed for any of the eight additional tumor cell lines, as growth rates of the pz-treated cells were similar to the growth rates of the DMSO-treated controls.

### 3.3. Dose-dependent effects of porphyrazines

**Figure 3:**
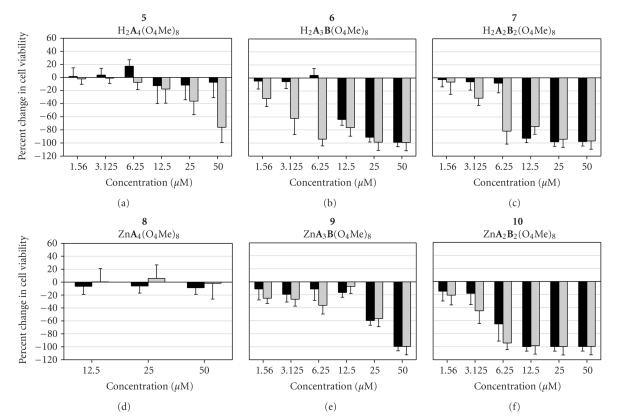
Percent cell viability, relative to DMSO control, of A549 (black bars) and WI-38 VA13 (gray bars) cells after a 72-hour exposure to pzs at varying concentrations. (Note that a 0% change in the percent cell viability correlates to cells which grew at the same rate as the control cells; negative values correlate to cells which grew at rates below those of the control cells.)

As described above, exposure to the M[pz(**A**_3_ **B**)] and M[pz(**A**_2_ **B**_2_)] pzs at concentrations of 50 *μ*M resulted in significant toxicity in both tumor and normal cells (Figure 2). To determine the toxicity limits of each pz, MTT assays were carried out in both A549 tumor and WI-38 VA13 normal cells at varying concentrations (1.56–50 *μ*M) for 72 hours. Figure 3 shows the dose-dependent results as percent cell viability, calculated relative to the viability of the control DMSO-treated cells. (Note that treated cells growing at an equivalent rate to the DMSO control cells would have a 0% change in cell viability; treated cells which grew slower and/or were less viable than the control cells would have a negative percent change in cell viability.)

With the exception of the M[pz(**A**_4_)] pzs, the thiotriethoxy(ethoxy)methyl-appended pzs were generally found to exhibit significant toxicity even at lower concentrations. Both of the M[pz(**A**_2_ **B**_2_)] pzs, **7** and **10**, caused nearly 100% cell killing at treatment concentrations of 12.5 *μ*M and above. Significant toxicity was still observed for both tumor and normal cell lines exposed to 6.25 *μ*M **10**; however, A549 tumor cells exposed to **7** at 6.25 *μ*M showed only very little toxicity (*∼*10% killed), while normal cells exposed to the same concentration resulted in nearly 80% of the cells dying. Slight toxicity was still observed in both normal and tumor cells exposed to the lowest concentration of **10** tested (1.56 *μ*M), while normal and tumor cells exposed to **7** at a treatment concentration of 1.56 *μ*M showed essentially normal growth upon a 72-hour exposure.

While **7** and **10** showed a somewhat similar dose-dependent proliferation/viability behavior, the two M[pz(**A**_2_ **B**_2_)] pzs, **6** and **9**, differed depending upon the identity of M. The H_2 _[pz(**A**_3_ **B**)], **6**, was found to exhibit a growth behavior very similar to that observed for its H_2_[pz(**A**_2_ **B**_2_)] analogue, **7**. Significant toxicity was found in both tumor and normal cells exposed to **6** at concentrations of 12.5 *μ*M and above for 72 hours; and similar to **7**, the normal cells showed selective toxicity at treatment concentrations of 6.25 *μ*M and below. However, unlike cells exposed to **7**, normal cells treated with **6** at 1.56 *μ*M still exhibited a moderate amount of toxicity (*∼*30% cell death). The Zn[pz(**A**_3_ **B**)] analogue, **9**, showed significantly less toxicity at the higher treatment concentrations (25 and 12.5 *μ*M) in both normal and tumor cells than that observed in cells treated with the free-base **6** at the same concentrations. However, slight toxicity (*∼*10–20% cell killing) was still observed for both tumor and normal cells exposed to low concentrations of **9,** whereas A549 tumor cells exposed to **6** at treatment concentrations of 6.25 *μ*M and below exhibited normal growth behavior.

Based upon the 50 *μ*M time-course plots in Figure 2, it was expected that the dose-dependence results of the M[pz(**A**_4_)] pzs (Figure 3) would generally show little toxicity. To this end, the free-base **5** exhibited selective toxicity towards WI-38 VA13 cells at concentrations of 25 *μ*M and higher upon a 72-hour exposure; at treatment concentrations of 12.5 *μ*M and below, any toxicity observed in either the tumor or normal cells fell within the experimental error limits for normal growth (i.e., within the error range for a 0% change in cell viability). Both normal and tumor cells exposed to the Zn[pz(**A**_4_)] pz, **8**, also exhibited normal growth behavior at all concentrations studied.

### 3.4. Photosensitizing effects of the porphyrazines

**Figure 4:**
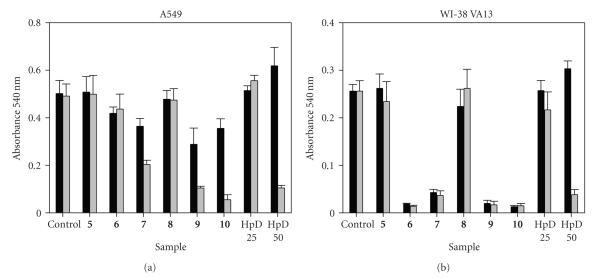
MTT results for A549 (a) and WI-38 VA13 (b) cells exposed to pz (25 *μ*M) or HpD (25 or 50 *μ*M) for 4 hours, followed by white light exposure for 0 (black bars) or 10 (gray bars) minutes. Control cells were treated with an amount of DMSO equivalent to 25 *μ*M pz.

In addition to the dark toxicity studies described above (Figures 2 and 3), light-dependent proliferation/viability studies were employed to determine if the compounds possessed additional photosensitizing properties. Due to the high levels of toxicity observed in the MTT dose-dependence studies (Figure 3), a shorter incubation time and a lower treatment concentration were employed for these compounds than used in the biological studies of previously reported pzs [15, 16]. Cell lines were treated with 25 *μ*M of **5–10** and 25 or 50 *μ*M HpD as a reference for 4 hours and were then exposed to white light for a period of 10 minutes. The microtiter plate serving as the dark control was completely wrapped in aluminum foil so that the cells would avoid any light exposure, but would still be exposed to any potential heat being generated by the bulbs of the X-ray illuminator during the 10-minute treatment period. The cells were incubated overnight following the light treatment, and MTT assays were performed after 24 hours. Figure 4 shows data comparing no light treatment versus cells exposed to 10 minutes of white light.

Both untreated (data not shown) and DMSO-treated cells were used as controls; similar results were obtained for both; therefore, Figure 4 shows only the DMSO-treated cells.

As expected, growth of the DMSO-treated cells was not adversely affected by the additional light exposure. However, unlike previously studied pzs [15, 16], three of the compounds reported here—**7**, **9**, and **10**—showed a distinct photosensitizing effect in the A549 tumor cells. It could not be determined if a similar effect occurred in the WI-38 VA13, due to the inherent toxicity of **7**, **9**, and **10** in the normal cells; absorption readings at the 0-minute light-exposure time point were extremely low for these three compounds in the WI-38 VA13 cell line (Figure 4). It is noted that, of the six pzs tested in this study, the three exhibiting the phototoxic effect in A549 cells correspond to the three compounds having the highest singlet oxygen quantum yield (*Φ*_Δ_). Accordingly, the compounds with relatively low *Φ*_Δ_ values—**5**, **6**, and **8**—did not show any appreciable phototoxic effect in either cell line, as viability levels remained steady with and without light treatment. Interestingly, no appreciable light effect was observed for either tumor or normal cells exposed to 25 *μ*M HpD (a dose that is two-fold below the typical clinical treatment dose); however, treatment of cells with HpD at the usual clinical dose, 50 *μ*M, results in substantial photo-induced cell death in both A549 and WI-38 VA13 cells, as shown in Figure 4.

**Figure 5:**
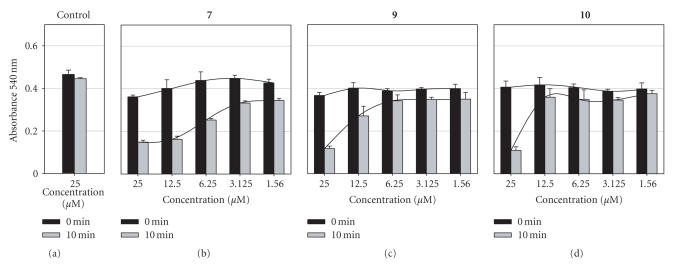
MTT results for A549 cells exposed to varying concentrations of pz or DMSO for 4 hours, followed by white light exposure for 0 (black bars) or 10 (gray bars) minutes. Line graphs are overlaid for clarity.

Additional dose-dependent phototoxicity studies were carried out for **7**, **9**, and **10** in A549 cells to determine if a light-dependent response could be observed for the pzs below the 25 *μ*M treatment dose employed in Figure 4. Cells were treated with the pzs at concentrations of 25–1.56 *μ*M for 4 hours and then exposed to either 0 or 10 minutes of white light. MTT assays were carried out after an overnight incubation (Figure 5). As expected from the results of Figure 4, Photofrin did not show any light-induced toxicity at treatment concentrations of 25 *μ*M or below (data not shown). However, significant phototoxicity was still observed in A549 cells treated with **7** at concentrations below 25 *μ*M upon a 10-minute white light exposure; a treatment dose of 12.5 *μ*M resulted in additional 60% of the cells being killed when exposed to light, while cells treated with 6.25 *μ*M **7** resulted in approximately 40% cell death, relative to the dark toxicity (i.e., 0 minute white light exposure). Even at the lowest treatment concentration of **7** studied, 1.56 *μ*M, a moderate amount (*∼*15–20% cell death) of toxicity was still observed. In contrast, both pzs **9** and **10** exhibited far less sensitivity than **7**. Moderate phototoxicity (*∼*30% cell death) was observed in A549 cells exposed to **9** at 12.5 *μ*M, but little effect was detected at doses of 6.25 *μ*M and below. Cells treated with **10** did not show any additional phototoxicity at doses below 25 *μ*M.

### 3.5. Cellular uptake of porphyrazines

**Figure 6:**
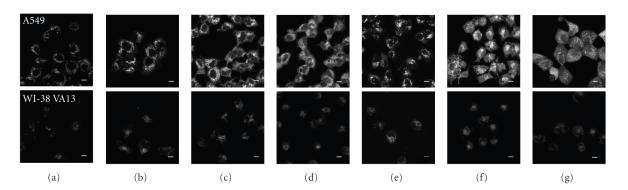
Cellular uptake of pzs using false-white confocal fluorescence microscopy images of A549 (top) and WI-38 VA13 (bottom) cells treated with 25 *μ*M agent: (a) DMSO control, (b) **5**, (c) **6**, (d) **7**, (e) **8**, (f) **9**, (g) **10**. Scale bar = 10 *μ*m.

Experiments were carried out to assess the cellular uptake and general localization behavior of each of the six pzs. Figure 6 presents the confocal microscopic images of A549 and WI-38 VA13 cells exposed to each compound for 4 hours at 25 *μ*M. Due to the rapid onset of toxicity caused by these compounds, as discussed above, the cells were treated at a lower concentration and for a shorter period of time than the conditions used in previous cellular uptake studies of the pzs [15].

Moderate autofluorescence was observed for the control (DMSO-treated) cells in both tumor and normal samples. All six of the pzs showed enhanced intracellular luminescence in A549 cells upon treatment with compound; each of the six compounds displayed similar fluorescence intensity and exhibited uniform staining of the cytoplasm with no uptake by the nucleus. In contrast, WI-38 VA13 cells showed little-to-no measurable increase in fluorescence upon treatment of the cells with the pzs. Significant vacuole formation was also observed in both tumor and normal cells exposed to **6**, **9**, and **10**.

## 4. Conclusions

Recent work has sought to study the structure-function relationships of the porphyrazines (pzs) in biological systems, in an effort to develop these compounds for use as therapeutic and diagnostic agents [15, 16]. Herein we continue our structure-function studies of the porphyrazines by examining a series of six new M[pz(**A**_n_ **B** ${}_{4\text{-}n}$)] porphyrazines, where M = H_2_ or Zn, **A** is [S((CH_2_)_2_O)_4_CH_3_]_2_, **B** is a fused 4,7-bis(isopropyloxy)benzo group, and n=2, 3, or 4 (Scheme 1). The thiotetra(ethylene glycol) monomethyl ether functional group is hydrophilic in nature, while the pz core is hydrophobic; thus for a given M, as the value of n is increased in the series, the resulting compounds progressively become more hydrophilic in character. Furthermore, by introducing zinc into the core of the pzs (**8**–**10**), the solubility of the compound and the singlet oxygen quantum yield are both enhanced, relative to their free-base analogues. The zinc pzs reported here are the first metallated pzs to be tested for their biological behavior. Concentration/time-dependent MTT proliferation/viability assays were carried out, both in the presence and absence of white light, in order to measure the dark and photoinduced toxicity of each pz in normal (WI-38 VA13) and tumor (A549) cell lines, and confocal microscopy was used to determine the cellular uptake and localization of each compound.

The dark toxicity studies of the six pzs revealed a dose-dependent response in which cellular toxicity increased in both normal and tumor cell lines as n was decreased (Figures 2 and 3). With the exception of **8**, all of the pzs were found to be toxic to normal cells at higher concentrations (25 *μ*M and above); and for the M[pz(**A**_3_ **B**)] and M[pz(**A**_2_ **B**_2_)] pzs, significant toxicity was also observed in the tumor cells at higher concentrations. In general, for a given concentration, the observed toxicity was found to be greater in the normal cells than in the tumor cells. Furthermore, in the case of the M[pz(**A**_3_ **B**)] and M[pz(**A**_2_ **B**_2_)] pzs, for a given n, toxicity was generally found to be higher for the free-base compounds than for the metallated pzs.

A particularly interesting result from the dark toxicity studies is the finding that **5** is selectively toxic to normal cells. As shown in Figure 2 and Table 1, **5** was found to be nontoxic across a number of different tumor cell lines and tumor types (both adenocarcinomas and squamous cell carcinomas), suggesting that this compound might possess multiple uses as a diagnostic agent. First, when attempting to establish a cell line derived from a human tumor (for purposes of preparing a cell line that will grow continuously in the laboratory), it is necessary to selectively remove and purify the tumor cells from the tumor sample removed from the patient, which contains a mixture of normal and cancerous tissue. This procedure is often very difficult and time-consuming; if **5** were added during this process, one could theoretically remove the normal cells more quickly, leading to faster purification of the desired tumor cell line. Second, the results of many patient biopsies are unclear as to whether a particular tissue sample is malignant or benign; in such unclear cases, one could treat the biopsy sample with **5** to verify the malignancy of the tissue.

The photo-induced toxicity studies (Figure 4) found further toxicity in A549 cells exposed to the pzs with higher singlet oxygen quantum yields—**7**, **9**, and **10**. These three compounds are the first members of the M[pz(**A**_n_ **B** ${}_{4\text{-}n}$)] subclass to exhibit photosensitivity [15, 16]. The result for **7** is particularly interesting, considering that previously studied H_2_[pz(**A**_2_ **B**_2_)] pzs all contained **A** groups that resulted in aggregation of the compound in aqueous media, thereby suppressing singlet oxygen generation. The result observed here suggests that we have achieved the proper hydrophobic/hydrophilic balance necessary for sufficient solubility in cellular environments, and this enhanced solubility is likely a key contributing factor in the observed photosensitivity of **7** at relatively low doses (Figure 5). Furthermore, the observation that **6** (**H**_2_A_3_B) did not exhibit a light-dependent toxicity, while **9** (**Zn**A_3_B) did show a response, suggests that the inherent singlet oxygen quantum yield of the free-base A_3_B structure may not be high enough to be applicable in PDT applications. Likewise, no photo-induced toxicity was observed for the M[pz(**A**_4_)] pzs, **5** and **8**, regardless of the identity of M. Therefore, the development of future pzs for PDT applications will focus mainly on members of the A_2_B_2_ (both free-base and metallated) and metallated A_3_B subfamilies. However, while members of the M[pz(**A**_4_)] and H_2_[pz(**A**_3_ **B**)] pz subfamilies may not provide any additional PDT activity, it is still important to study pzs belonging to these subfamilies in an effort to discover new selectively toxic antitumor agents.

Confocal fluorescence microscopy images revealed that all six of the compounds prepared in this study were preferentially incorporated into the A549 tumor cells and localized in the cytoplasm. The results of the cellular uptake studies further suggest that **8** is a particularly promising imaging agent since it was found to be nontoxic upon exposure to both tumor and normal cells and was selectively taken up by tumor cells. As mentioned above, vacuole formation was observed in both the tumor and normal cells upon exposure to **6**, **9**, and **10**, suggesting that these compounds may be inducing terminal differentiation, a specialized form of apoptosis [21, 22]. Future work will more closely examine the mechanism by which the pzs in this report (**5**–**7**, **9**, **10**) cause cell death. By determining the role of the pzs in cell destruction, we may be able to slightly alter the structure of the compounds in order to minimize toxicity (while maintaining the proper pz solubility and selective tumor uptake), thus leading to many other potential imaging agents from this class of compounds.

The results of this study are the first steps in the search for useful tumor specific imaging agents and compounds for use in purifying cell lines from clinical samples. If the issue of nonselective toxicity can be overcome, the path to developing porphyrazines as useful antitumor drugs (both traditional and PDT-activated) will be opened up.

## Figures and Tables

**Table 1:** Percent cell viability of various cell lines exposed to 50 *μ*M **5** for 72 hours, relative to DMSO-treated cells.

Cell Line	Cell Line Description	Percent Cell Viability (%)
SCC016	Human tongue squamous cellcarcinoma	91.2±19.2%
SCC040	Human tongue squamous cellcarcinoma	95.3±23.8%
SCC056	Human tongue squamous cellcarcinoma	94.8±20.5%
SCC114	Human floor of mouth squamous cellcarcinoma	78.2±17.0%
SCC116	Human alveolar ridge squamous cellcarcinoma	102.0±10.2%
BT-20	Human breast adenocarcinoma	118.4±4.5%
MCF-7	Human breast adenocarcinoma	77.7±19.6%
T-47D	Human breast adenocarcinoma	106.4±8.4%
A549	Human pulmonary adenocarcinoma	92.7±23.6%
WI-38 VA13	Human embryonic fibroblast	20.9±23.3%
